# Genome-wide association study as a powerful tool for dissecting competitive traits in legumes

**DOI:** 10.3389/fpls.2023.1123631

**Published:** 2023-08-14

**Authors:** Pusarla Susmitha, Pawan Kumar, Pankaj Yadav, Smrutishree Sahoo, Gurleen Kaur, Manish K. Pandey, Varsha Singh, Te Ming Tseng, Sunil S. Gangurde

**Affiliations:** ^1^ Regional Agricultural Research Station, Acharya N.G. Ranga Agricultural University, Andhra Pradesh, India; ^2^ Department of Genetics and Plant Breeding, College of Agriculture, Chaudhary Charan Singh (CCS) Haryana Agricultural University, Hisar, India; ^3^ Department of Bioscience and Bioengineering, Indian Institute of Technology, Rajasthan, India; ^4^ Department of Genetics and Plant Breeding, School of Agriculture, Gandhi Institute of Engineering and Technology (GIET) University, Odisha, India; ^5^ Horticultural Sciences Department, University of Florida, Gainesville, FL, United States; ^6^ Department of Genomics, Prebreeding and Bioinformatics, International Crops Research Institute for the Semi-Arid Tropics, Hyderabad, India; ^7^ Department of Plant and Soil Sciences, Mississippi State University, Starkville, MS, United States; ^8^ Department of Plant Pathology, University of Georgia, Tifton, GA, United States

**Keywords:** breeding, genomic selection, linkage, mapping, phenotyping, protein

## Abstract

Legumes are extremely valuable because of their high protein content and several other nutritional components. The major challenge lies in maintaining the quantity and quality of protein and other nutritional compounds in view of climate change conditions. The global need for plant-based proteins has increased the demand for seeds with a high protein content that includes essential amino acids. Genome-wide association studies (GWAS) have evolved as a standard approach in agricultural genetics for examining such intricate characters. Recent development in machine learning methods shows promising applications for dimensionality reduction, which is a major challenge in GWAS. With the advancement in biotechnology, sequencing, and bioinformatics tools, estimation of linkage disequilibrium (LD) based associations between a genome-wide collection of single-nucleotide polymorphisms (SNPs) and desired phenotypic traits has become accessible. The markers from GWAS could be utilized for genomic selection (GS) to predict superior lines by calculating genomic estimated breeding values (GEBVs). For prediction accuracy, an assortment of statistical models could be utilized, such as ridge regression best linear unbiased prediction (rrBLUP), genomic best linear unbiased predictor (gBLUP), Bayesian, and random forest (RF). Both naturally diverse germplasm panels and family-based breeding populations can be used for association mapping based on the nature of the breeding system (inbred or outbred) in the plant species. MAGIC, MCILs, RIAILs, NAM, and ROAM are being used for association mapping in several crops. Several modifications of NAM, such as doubled haploid NAM (DH-NAM), backcross NAM (BC-NAM), and advanced backcross NAM (AB-NAM), have also been used in crops like rice, wheat, maize, barley mustard, etc. for reliable marker-trait associations (MTAs), phenotyping accuracy is equally important as genotyping. Highthroughput genotyping, phenomics, and computational techniques have advanced during the past few years, making it possible to explore such enormous datasets. Each population has unique virtues and flaws at the genomics and phenomics levels, which will be covered in more detail in this review study. The current investigation includes utilizing elite breeding lines as association mapping population, optimizing the choice of GWAS selection, population size, and hurdles in phenotyping, and statistical methods which will analyze competitive traits in legume breeding.

## Introduction

The term legume originated from the Latin word “legumen”, which denotes “seeds harvested in pods”. During the Neolithic Revolution, which marked the beginning of human farming methods, farmers were accompanied by legumes that belong to the family Fabaceae. It is acknowledged that inadequate protein-energy intake and micronutrient deficits are two of the primary causes of undernutrition. Legumes play a minor but significant role in our food system. They are the superior economical dietary solutions due to their rich protein content (17%–30%) and relevant nutritional richness compared to expensive food sources containing animal-based protein and dairy products that may be difficult to obtain in situations where there is food insecurity (Marinangeli et al., 2017).

Compared with cereals, legumes provide a substantial quantity of protein throughout the complete plant, notably in grains. The incorporation of leguminous crops in cropping systems enabled an enhancement in soil quality (Hasanuzzaman et al., 2020). Legumes’ ability to fix atmospheric nitrogen in symbiotic relationships with soil bacteria such as Rhizobium and Brady rhizobium minimizes the requirement for chemical fertilizers during crop growth and contributes to a reduction in greenhouse gas emissions like nitrous oxide (N_2_O) and carbon dioxide (CO_2_). In addition, they can help to reduce the utilization of fossil-based energy inputs in the chain of agriculture and food production by infusing high-quality organic matter, facilitating nutrient circulation, and promoting water retention in the soil (Stagnari et al., 2017). Legumes are rich in nutraceuticals, such as vitamin B6, calcium, magnesium, sodium, zinc, copper, and manganese. Thus, it is crucial to expand the genetic background and foster the breeding of legume crops, which will serve the needs of the growing human population under changing climatic conditions. Therefore, it is essential to come up with high-yielding cultivars that have enhanced resistance to diseases, higher nitrogen fixation ability, and tolerance to abiotic and biotic stresses, which can be achieved using biotechnological and genomics-assisted breeding approaches.

Genome-wide association study (GWAS) is an effective technique for determining the genes underlying a particular trait. To accomplish this, it is ideal to assess the genomic regions where genotypic and phenotypic variations are correlated with each other. In comparison to standard biparental populations, GWAS offers greater mapping precision for detecting interactions among molecular markers and desirable characteristics in a variety of crops (Liu et al., 2016; Cui et al., 2017). It has become a vital tool in agricultural genetics due to its techniques that build upon the mixed linear model (MLM) framework and deliver radically improved computational speed and statistical power.

Furthermore, improvements can be applied in fields like omic-wide association studies, which utilize GWAS techniques to analyze relationships among desirable morphological traits and other kinds of omics data that include transcriptomic or metabolomic. GWAS requires structuring the population of diverse panels to estimate genetic distinction and minimize the detection of spurious connections (Sul et al., 2016). Breeders can develop new varieties owing to recent innovations in NGS applications and technologies that enable advanced tools to characterize genetic variation at a high resolution (Gali et al., 2019). The ultimate objective of this review is to quantify the genetic diversity, GWASs, and other related aspects or techniques that could be used to break the plateau of yield in legume crop production and can be utilized for further crop improvement.

## Mapping population in association studies

Association mapping (AM), an alternative to QTL mapping, is dependent on linkage disequilibrium (LD) and uses collections of genotypes with known or unknown ancestry that have a significant degree of genetic variation due to hundreds of recombination cycles. The ultimate goal of association studies is to find a strong correlation between a genome-wide DNA marker and an interesting attribute that can be highly useful in marker-assisted selection for crop development. GWAS and candidate gene (CG)–based analysis are two important approaches to AM.

**Figure 1:**
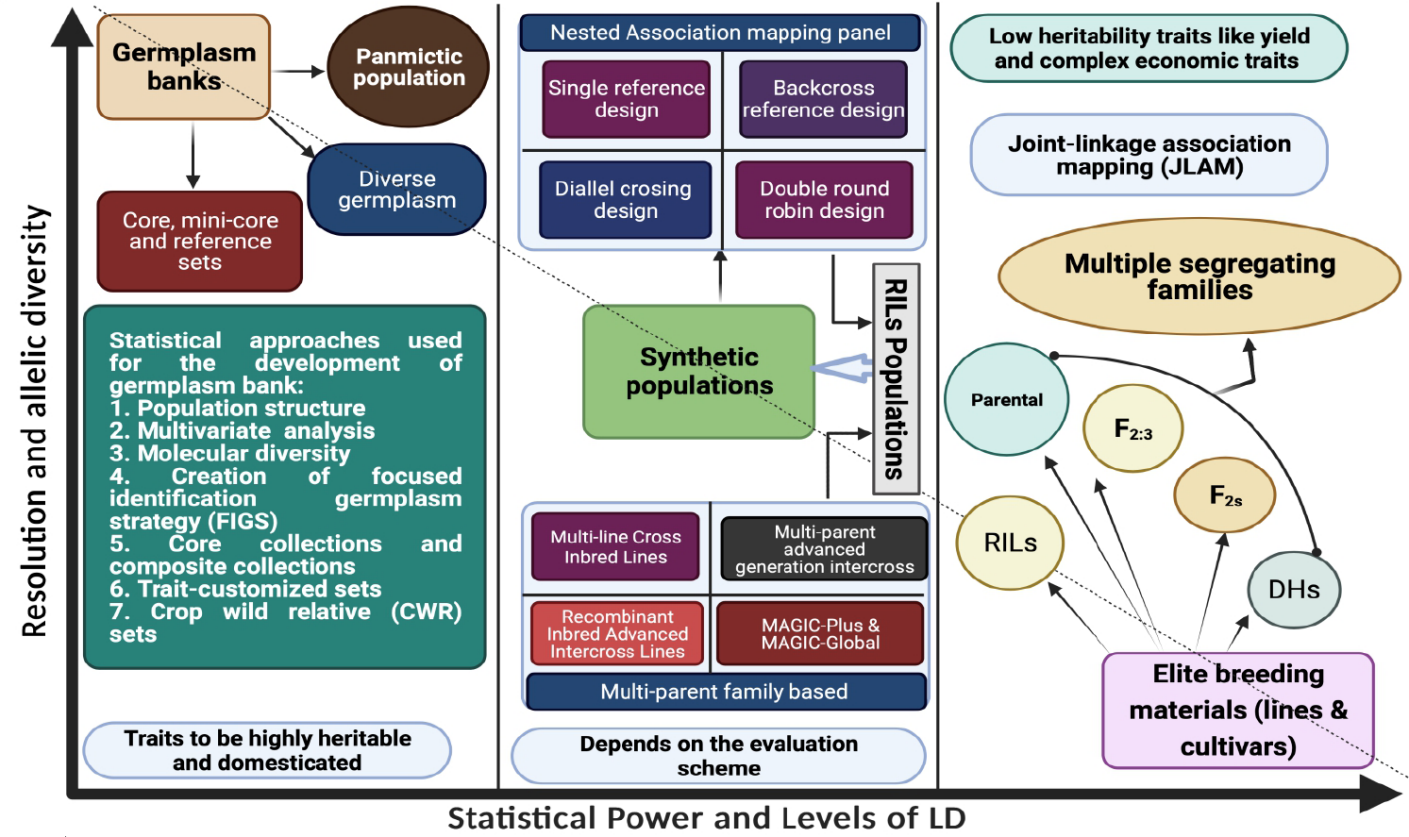
Types of mapping population used in GWAS studies along with their brief properties.

The creation of a mapping population that will be tested for the marker–trait relationship is a prerequisite for the GWAS. Both broad-based natural populations and narrow-based breeding populations can be utilized as the mapping population for GWAS (**Figure 1**). The sort of mapping population needed for the success of GWAS hangs significantly on the mode of pollination (inbreeding or outbreeding) of the plant species. Both natural diverse germplasm panels and family-based breeding populations can be used for this. Among the breeding population, both biparental and multiparental mapping populations such as Multiparent Advanced Generation Inter-Cross ([MAGIC), Multiline Cross Inbred Lines (MCILs), Recombinant Inbred Advanced Intercross Lines (RIAILs), Nested Association Mapping (NAM), and Random Open- parents Association Mapping (ROAM) are being used for AM in several crop plants. Populations such as doubled haploid NAM (DH-NAM), backcross NAM (BC-NAM), and advanced backcross NAM (AB-NAM) that are modifications of NAM have also been used in recent times. The selection of the mapping population should be taken care of enough to avoid the false-positive marker–trait association. Because of the problematic inconsistent phenotyping scores of segregating lines over the years and location, heterozygote segregating individuals should not be included with the inbred lines as one population when creating the AM panel. When significant features like days to bloom and maturity are influencing the target trait, extreme genotypes should be eliminated from the AM panel for proper scoring of trait data (Kulwal and Singh, 2021). Each population has unique virtues and flaws, which will also be discussed further in the review study.

## Natural population and elite breeding lines as association mapping population

Any naturally occurring panmictic population with a significant history of recombination events can undergo AM. Utilizing hundreds of recombination events makes it simple to do an LD analysis of the target characteristic. These populations, however, are not appropriate for QTL mapping. When a germplasm accession collection represents the natural population, it may be a core collection or a sample that is more resilient to environmental changes. The population is excellent for assessing the QTLs for rare alleles that can help develop elite breeding lines or highly heritable domestic features. QTLs for some agronomically key characteristics have been uncovered in germplasms of several crops using GWAS, such as in 135 pea accessions (Gali et al., 2019), 366 sesame accessions (Cui et al., 2017), and 119 accessions in rice (Pawar et al., 2021).

The cultivars and lines created by a deliberate breeding program are known as elite inbred lines. These lines are unbreakable and can be maintained by numerous researchers in various places to identify QTLs using an AM panel. For instance, two AM panels of maize having 306 dent corn and 292 European flint corn inbred lines were individually assessed using single-nucleotide polymorphism (SNP) markers in the cold and control growth chamber conditions to identify genes related to cold tolerance (Revilla et al., 2016). For GWAS research in sorghum, AM panels of 377 tropical accessions from various geographic and climatic zones, significant U.S. breeding lines, and the wild species have been brought together to be used as AM panels (Casa et al., 2008). GWASs in legumes mostly include the natural populations and elite advanced breeding lines (**Table 1**), whereas GWAS using artificial mapping populations is more or less a recent phenomenon, and they are still underway.

**Table 1:** GWAS studies for various traits in different leguminous crops.

Crop	Mapping population	Traits	QTLs/Marker trait associations	References
Gram	132 varieties and Advanced Breeding Lines (ABLs)	Yield traits	38 MTAs (marker trait association)	Li et al., 2018
	192 desi & kabuli accessions	Seed weight	8 MTAs	Bajaj et al., 2016
	182 diverse genotypes	Phenological, physiological and yield traits	14-34 MTAs in different environment condition	Jha et al., 2021
	75 ABLs	Fusarium wilt	3 MTAs	Jha et al., 2021
	165 chickpea genotypes	resistance to *Ascochyta rabiei*	30 MTAs	Farahani et al., 2022
	280 accessions	Grain Nutrient and Agronomic Traits	20 and 46 MTAs for grain nutrient and agronomic traits, respectively	Srungarapu et al., 2022
Arhar	Diverse collection of 142 pigeonpea lines	Flowering related traits	22MTAs	Kumar et al., 2022
	Pangenome based on 89 accessions	9 agronomic traits	229 MTAs	Zhao et al., 2020
Faba beans	481 elite breeding lines	Agronomic Traits	30 MTAs	Keller et al., 2020
Lentil	188 lines of the USDA Lentil Core Collection	Pea aphid	15 candidate genes	Das et al., 2022
Pea	135 pea accessions	Agronomic and Seed Quality Traits	251 MTAs	Gali et al., 2019
	135 pea accessions	Heat and Drought Adaptive Traits	15 MTAs	Tafesse et al., 2021
Mungbean	127 test genotypes	Mungbean yellow mosaic India virus resistance	15 MTAs	Singh et al., 2020
	95 cultivated mung bean genotypes	Seed Mineral content	43 MTAs	Wu et al., 2020
Blackgram	100 diverse genotypes	Agronomic traits	42 QTLs	Singh et al., 2022
	99 diverse genotypes	Agronomic traits	83 MTAs	Nkhata et al., 2021
Soybean		Protein, Oil, unsaturated fatty acid, oleic acid	SNP	(Hwang et al., 2014; Zhang et al., 2019; Zhao et al., 2019; Liu et al., 2020)
		Nematode resistance, Iron deficiency and Canopy wilt, brown stem rot, Diseases resistance	SNP	(Butenhoff, 2015; Vuong et al., 2015; Chang et al., 2016; Rincker et al., 2016; Zhang et al., 2017a; Zhang et al., 2017b; Zatybekov et al., 2018; Do et al., 2019; Ravelombola et al., 2019; Tran et al., 2019; Che et al., 2020; Lin et al., 2020)
		Salt tolerance, Flood tolerance, Drought tolerance, Water Use Efficiency	SNP	(Dhanapal et al., 2015; Zeng et al., 2017; Chen et al., 2018; Khan et al., 2018; Yu et al., 2019; Assefa et al., 2020)
		Agronomic trait	SNP	(Wen et al., 2015; Zhang et al., 2015; Contreras-Soto et al., 2017; Yan et al., 2017; Zhang et al., 2021; Zatybekov et al., 2017; Pan et al., 2018; Hu et al., 2019a; Li et al., 2019; Kim et al., 2020)
		Physiological traits	SNP	(Sui et al., 2020; Wang et al., 2020; Yang et al., 2020)
Groundnut	170 genotypes	Quality traits	SNP	(Shaibu et al., 2019a)
	125 ICRISAT groundnut mini core collection	Physiological traits	SNP	(Shaibu et al., 2019b; Shaibu et al., 2020)
	158 peanut accessions; 195 peanut accessions	Agronomic traits	SNP	(Zhang et al., 2017c; Wang et al., 2019)
	120 genotypes	Disease resistance	SNP	(Zhang et al., 2019; Zhang et al., 2020)
	249 peanut accessions	Abiotic stress tolerance	SNP	(Zou et al., 2020)
Chickpea		Agronomic traits	SNP	(Bajaj et al., 2015a; Bajaj et al., 2016; Kujur et al., 2015a; Upadhyaya et al., 2015; Upadhyaya et al., 2017; Basu et al., 2018; Orsak et al., 2019; Fayaz et al., 2022; Srungarapu et al., 2022)
		Abiotic stress tolerance	SNP	(Thudi et al., 2014; Li et al., 2018; Kohli et al., 2020; Ahmed et al., 2021; Kalve et al., 2022)
		Physiological traits	SNP	(Basu et al., 2019)
		Quality traits		(Upadhyaya et al., 2016a; Upadhyaya et al., 2016b; Parida et al., 2017; Samineni et al., 2022)
		Biotic stress resistance		(Li et al., 2017; Agarwal et al., 2019; Agarwal et al., 2022; Farahani et al., 2022; Raman et al., 2022)
Beans		Agronomic traits/ Quality	SNP	(Warsame et al., 2019; Rasool et al., 2022)
		Abiotic stress	SNP	(Ali et al., 2016; Li et al., 2017; Hu et al., 2019b; Breria et al., 2020; Abou-Khater et al., 2022; Maalouf et al., 2022; Sallam et al., 2022)
		Biotic stress	SNP	(Faridi et al., 2021)
Lentils		Agronomic traits	SNP, SSR	(Kumar et al., 2018a; Kumar et al., 2018b; Singh et al., 2019; Karthika et al., 2021; Neupane et al., 2022)
		Quality traits, Seed quality	SNP	(Khazaei et al., 2017; Khazaei et al., 2018; Johnson et al., 2021; Hang, 2022; Puspitasari et al., 2022)
		Biotic stress	SNP	(Banoo et al., 2020; Gela et al., 2021)
		Abiotic stress	SSR	(Singh et al., 2017; Kumar et al., 2019; Ma et al., 2020)

## Biparental mapping population and association mapping

Recombinant inbred lines (RILs) and Near Isogenic Lines (NILs) are the most used biparental population, usually used for linkage mapping or Quantitative Trait Locus (QTL) or QTL mapping. Whereas the power of QTL identification is higher in linkage mapping as compared to AM, the resolution has a reverse relationship with both mapping schemes. The concept of joint linkage AM (JLAM) was introduced to fully exploit the capabilities of both mapping methods. JLAM uses either a biparental population set or one or more multiparental AM panels, or two sets of genotypes consisting of germplasm and biparental mapping populations, which are genotyped utilizing the same set of markers (Myles et al., 2009; Lu et al., 2010; Reif et al., 2010 and Wurschum et al., 2012). Hence, JLAM is also recognized as integrated mapping that identifies more significant marker–trait associations and increases the power of AM. Using JLAM (by combining germplasm accessions and full-sib F2 population of a bioenergy crop Shrub willow (*Salix* sp.) identified several major QTLs along with QTL hotspots (Carlson et al., 2019). Several studies using JLAM include QTL identification and CG identification for drought tolerance in maize (Lu et al., 2010), pleiotropic QTLs for silique length and seed weight in rapeseed (Li et al., 2014), and the epistatic QTLs for agronomically important characters in sugarbeet (Reif et al., 2010). Recent studies claim that regulating population structure and addressing rare alleles can be accomplished through cofactors and a demographic effect accounting for JLAM, which enhances the predictive power of the methods (Wurschum et al., 2012).

## Multiparent mapping population for GWAS

The multiparent populations include several founder parents, which reflect wider genetic diversity. Hence, in AM studies, the use of multiparent mapping populations helps limit the demerit of recombination frequency in biparental populations. Multiparent mapping populations provides tools to control population structure and balance allele frequencies. The historical and artificial recombinational events of the multiparent mapping populations such as NAM and MAGIC populations and their derivatives increase the efficiency of QTL identification in AM. Because of the controlled crosses, NAM population has higher power because of maximized population structure and minimal familial relatedness and accumulated frequency of rare alleles. The population facilitates cost-effective GWAS and allows the perpetual sharing of the NAM panel with global researchers.

To generate sets of RILs, NAM populations can be developed using reliable mating strategies such as diallel mating, NCD-II (North Carolina design II), eight-way cross, and single/double round robin. NAM population was first developed in maize using RILs developed from a diverse set of parents. Twenty-five diverse families in maize were used to develop 5,000 RILs that were evaluated for southern leaf blight disease resistance (Kump et al., 2011), and the wide diversity helped in the identification of 32 QTLs for the trait. A NAM population was developed using 23 different inbreds of barley in a twofold round-robin design to identify QTLs and CGs for grain morphology (Shrestha et al., 2022). NAM population has been established in both autogamous and allogamous species such as barley, rice, wheat, sorghum, and maize (Maurer et al., 2015; Bajgain et al., 2016). Several modifications of NAM, such as DH-NAM, BC-NAM, and AB-NAM, have also been used in recent times. An AB-NAM of barley consists of 796 BC_2_F_4:6_ lines, which were derived from 25 wild barley accessions by backcrossing to the cultivar Rasmusson (Nice et al., 2016). Using 384 SNP markers, the AB-NAM population with minimal undesirable and unadapted characteristics of the wild barley parents was genotyped and encountered 10 QTLs for grain protein content (Nice et al., 2016).

A MAGIC population is created by a group of RILs from a complex cross or a group of crosses with numerous parents. Multiple rounds of recombination occur as these populations mature, improving the accuracy of desirable recombination and desirable alleles, thereby increasing the resolution of QTL mapping. With the aid of single seed descent (SSD), highly homozygous lines will be developed to establish the MAGIC population. To develop MAGIC populations for wheat and rice that can be deployed for QTL mapping, indica and japonica lines have been adopted. Seven cycles of SSD selfing resulted in 305 F8 lines in cowpea (*Vigna unguiculata*) (Huynh et al., 2018). In the MAGIC indica rice population, 400 lines from S2 bulk were chosen on the basis of agronomic attributes and evaluated in mega-environment trials to select elite lines (Bandillo et al., 2013).

## New high-throughput genotyping technologies for plants

The molecular markers are being progressively used to expedite breeding efforts in the post-genome sequencing era. Modern plant breeding is shifting from classical breeding to molecular breeding, where various genotyping technologies are being used for the discovery of molecular markers. In the last decade, a huge number of molecular markers were used for structural analysis of large germplasm populations to understand the diversity and use in GWAS. The whole-genome sequencing for most of leguminous crops has already been completed. Chromosome-level genomes are completed for most of the leguminous crops (Varshney et al., 2013). In the pre-genome sequencing era, the simple sequence repeat (SSR) markers were very powerful and potentially used for GWAS analysis. SSRs are tandem repeats highly polymorphic, abundant, co-dominant, and distributed throughout the genome. However, SSR markers are very laborious and time-consuming when compared with modern genotyping platforms such as double-digest restriction site–associated DNA sequencing (ddRAD-Seq) or specific locus amplified fragment sequencing (SLAF-Seq), whole-genome resequencing (WGRS), genotyping-by-sequencing (GBS), SNP-chip arrays, diversity array technology (DArT) array technology. With Illumina, gigabases of DNA sequencing data may be generated in a short period and cost-effectively in the NGS era (Bentley et al., 2008), Roche (Rothberg and Leamon, 2008), and AB-SOLiD (Pandey et al., 2008).

Molecular markers have become crucial components in molecular breeding over the past 2 years (Nadeem et al., 2018; Horst and Wenzel, 2007; Eathington et al., 2007). Molecular breeding has gained popularity and has been accepted by plant scientists because of its rapid and precise results for germplasm classification, back cross-breeding, and marker-assisted selection (Kumar et al., 2011; Nair and Pandey, 2021). A plethora of studies has been done using molecular markers (Kujur et al., 2015a; Wu et al., 2010; Song et al., 2013; Deokar et al., 2014; Shao et al., 2022). Different types of markers have been used for genotyping of legume, which includes rapid amplified polymorphic DNA (RAPD) (Doldi et al., 1997; Thompson et al., 1998; Iruela et al., 2002; Talebi et al., 2008), amplified fragment length polymorphism (AFLP) (Nguyen et al., 2004; Singh et al., 2008; Ude et al., 2002), inter-SSR (ISSR) (Yadav et al., 2014; Bhagyawant and Srivastava, 2008; Iruela et al., 2002; Souframanien and Gopalakrishna, 2004), and SSR (Saxena et al., 2010; Choudhary et al., 2012; Zavinon et al., 2020).

However, continuous improvement of next-generation sequencing (NGS) technologies in recent years has made it cost-effective and accessible for any crop, including legumes (Poland et al., 2012). Reference genome sequencing has been completed in some legume crops like soybean, pigeon pea, groundnut, cowpea, chickpea, and common bean (Afzal et al., 2022; Salgotra and Stewart, 2022). The currently available NGS technologies sequence each molecular or base pair of the DNA of any organism and make it feasible for us to identify the number of SNP markers with high precision and in a very short period (Liew et al., 2004). Although SNPs are biallelic and their polymorphism information is much lower compared to SSRs, they cover a significantly large part of the genome, which makes them markers to go for GWASs. In the last decade, a plethora of genotyping studies were carried out using SNPs in chickpea (Kujur et al., 2015b; Gaur et al., 2012; Hiremath et al., 2012; Deokar et al., 2014), pigeon pea (Raju et al., 2010; Singh et al., 2016; Arora et al., 2017), groundnut (Varshney, 2016; Pandey et al., 2017; Abady et al., 2021), soybean (Wu et al., 2010; Song et al., 2013; Shao et al., 2022), and other legume crops (Bohra et al., 2021; Shilpa and Lohithaswa, 2021).

## PCR-based genotyping methods

Amplification of DNA segments with PCR leads to the development of multiple genotyping methods. If the primers in a PCR reaction include the variation of interest, then it is called as allele-specific PCR. Allele-specific markers are generally used during foreground selection during marker assisted selection. PCR-Restriction Fragment Length Polymorphism (RFLP) is another method of PCR-based genotyping (Saiki et al., 1985), where the genomic region of interest is PCR-amplified using the markers and then digested with restriction enzymes specifically recognize a DNA sequence, so that the digested product can produce alleles of different size, which can distinguish among the individuals. Microsatellites or short tandem repeat polymorphisms are ideal markers for PCR-based genotyping as the length of the amplified DNA fragment varies based on repeats of microsatellites in the genome (Weber and May, 1989). Before NGS technologies, a variety of DNA-based markers have been developed and used for genotyping, for instance, RAPD, SSRs (Hong et al., 2021), ISSRs, and AFLP. Among them, SSRs were most widely used in genotyping and genetic mapping studies. PCR-based genotyping methods are cheaper as compared to NGS technologies. However, the PCR-based genotyping methods are laborious and not highly efficient as NGS-based genotyping. The NGS-based genotyping includes restriction digestion of DNA and sequencing of libraries.

## Double-digest restriction site–associated DNA

Although the SSRs are a potent marker system because of high reproducibility, co-dominance, and polymorphism, it is time, therefore, to generate the thousands of genome-wide SNP markers, restriction-sites associated with DNA sequencing (RADSeq) for large populations to study population genetics and genetic dissection of complex traits (Davey and Blaxter, 2011). However, in RADSeq, ~30%–50% of data were discarded because of repeated variable sites. The more reliable technique of double-digest restriction site–associated DNA sequencing (ddRAD-Seq) was developed to boost the efficiency (Peterson et al., 2012). The ddRAD-seq simultaneously uses two restriction enzymes to decrease the genome entanglement and library preparation cost by five-folds and can capture the genomic regions in hundreds of thousands for enhanced representation of the genome. It was successfully used in genetic mapping studies in peanut to map the QTLs for late leaf resistance and plant type–related traits (Zhou et al., 2014; Zhou et al., 2016). The ddRAD-seq was further advanced to reduce the repetitive DNA sequences, and the optimized version of ddRAD-Seq was developed called SLAF-Seq. The steps in SLAF-Seq are the same as in ddRAD-Seq. The DNA fragments are optimized for even distribution and to reduce the repetitive sequences. However, both technologies do not cover the whole genome (Sun et al., 2013).

## Genotyping-by-sequencing

GBS is a robust genotyping technology used for SNP discovery for a multitude of applications (Elshire et al., 2011). It is a variation of ddRAD-seq, first discovered in maize and barley used for genotyping recombinant inbred line populations. In GBS, methylation-sensitive restriction enzymes play a vital role in DNA digestion that lessens the genome complexity while constructing the sequence libraries. The genomic areas that are difficult to access to contemporary sequencing techniques can be captured by GBS. The GBS was efficiently used in groundnut for trait mapping (Jadhav et al., 2021) and diversity analysis (Khera et al., 2013). Pandey and co-workers (2014) performed GWAS analysis using SSR and GBS-based SNP genotyping data to identify the SNPs associated with aflatoxin contamination and agronomic traits in groundnut. GBS was used for genotyping cultivated and wild accessions of chickpea to discover 82,489 SNPs used for diversity, population structure, and LD analysis (Bajaj et al., 2015a; Kujur et al., 2015a). A total of 3,187 SNPs were used to reveal the genetic cluster associated with black-seeded genotypes of chickpea. GBS was also used for genotyping biparental populations in trait mapping studies to identify the QTLs for sterility mosaic disease (Saxena et al., 2017a), fusarium wilt (Saxena et al., 2017b), and fertility restoration (Saxena et al., 2018) in pigeon pea. In chickpea, drought tolerance–related “*QTL-hotspot*” was discovered with 743 SNP loci (Jaganathan et al., 2015), and 3,228 SNP loci were used for mapping and identification of CGs of seed traits (Verma et al., 2015). The multiplex sequencing strategy by using adapter sequences makes GBS very inexpensive. However, it produces more missing calls, and imputations are highly recommended during quality analysis. However, GBS is also incomplete, as its sequencing covers only a limited genome (~2.5%). GBS has replaced the previous genotyping markers, i.e., RAPD, ISSR, and SSRs, as it requires less time and labor and is highly cost-effective. GBS technology has been done in legumes like chickpea and soybean (Shingote et al., 2022; Torkamaneh et al., 2021; Iquira et al., 2015; Bajaj et al., 2016; Sudheesh et al., 2021; Kujur et al., 2015b; Verma et al., 2015).

## Diversity array technology

The polymorphic DNA segments called DArT markers in a genome are recognized through differential hybridization on a diversity genotyping array (Jaccoud et al., 2001). DArT is a very cost-effective whole-genome DNA fingerprinting tool for a variety of genetic analyses. It is a high-throughput sequence–independent technology that combines restricted-based hybridization and PCR. It is a very efficient marker system that can discover thousands of polymorphic sites in a very short time in any crop species. DArT is very popular in terms of high genome coverage, speed, reproducibility, and reliability (Aitken et al., 2014). Furthermore, polymorphic fragment calling does not require the reference genome. The DArT technology can be effectively used for genomic selection (GS) (Varshney et al., 2017) and marker-assisted selection (Stojaowski et al., 2011). However, the DArT markers are redundant due to clones with common sequences. Therefore, the presence of redundancy and markers with low frequencies (~41%) may affect the statistical analysis that is needed to filter out. DArT procedure includes generating a diversity panel followed by genotyping using a diversity panel. The first-ever genetic map of any legume crop was designed using DArT technology by Yang and coworkers (2011) in pigeon pea. A biparental population (F_2_) was screened using 554 DArT markers. Olukolu et al. (2012) used the DArT marker technology for genetic diversity assessment of 124 accessions of groundnut representing 25 countries of Africa. Roorkiwal et al. (2014) used the DArT markets to diversify the 10 *Cicer* species, including 94 genotypes. Aldemir and coworkers (2017) used an advanced version of DArT technology, i.e., DArt sequencing (DArTseq), for the identification of QTL for iron content in lentil seeds. DArTseq is also a hybridization-based technology but combines with NGS and provides a much simpler form of sequencing than DArT (Courtois et al., 2013; Aldemir et al., 2017). Ates (2019) estimated the genetic diversity of 94 lentil landraces with DArt-based 19,383 SNPs.

## SNP arrays

NGS technologies discovered an ample number of SNP markers because the demand for high-throughput genotyping has increased. The hybridization-based microarray or SNP arrays are very popular in genetic mapping, diversity analysis, and population genomics (You et al., 2018). SNP array or DNA microarray are highly polymorphic and use designed probes hybridized with fragmented DNA, which determines the alleles of all the SNP positions for hybridized DNA samples (LaFramboise, 2009). On the basis of the density, the SNP arrays can be divided into high-density (>50K), mid-density 5–10K), and low-density (>5K) SNP arrays. High-density SNP arrays can be used for high-density genetic mapping, GWAS, and population genomics studies. Mid-density assays can be used in GS because a few thousand SNPs are enough based on the genome size of the individual. However, the low-density SNP arrays can be used for foreground and background selection during marker-assisted selection and several breeding purposes. For instance, the quality control panel of rice is a low-density SNP array (25 SNPs), highly used for F1 confirmation, hybrid purity testing, and DNA fingerprinting in rice (Ndjiondjop et al., 2018). SNP arrays have been efficiently developed in several crops for genotyping, such as maize (600K SNP array) (Unterseer et al., 2014), apple (480K SNP array) (Bianco et al., 2016), and rice (700K SNP array) (McCouch et al., 2016). In leguminous crops such as peanut, the SNP Arachis array with 58K SNPs (Pandey et al., 2017) was very successful for genetic mapping (Pandey et al., 2020) and association analysis (Gangurde et al., 2020) for several traits. In pigeon pea, 56K Axiom Cajanus SNP Array and chickpea 11K Axiom Cicer SNP Array were developed (Roorkiwal et al., 2018). However, they are fixed and may not capture all recombination or diversity in an association panel, which are the limitations of SNP arrays. For instance, for genotyping a multi-parent population such as MAGIC or NAM, the whole-genome resequencing–based genotyping is helpful to capture maximum recombination regions.

## Whole-genome resequencing

Advanced NGS technologies reduced per-sample sequencing cost, and WGRS-based genotyping was used for many populations to identify the presence of absence variations for genome-wide association analysis. WGRS can be carried out at high depth or low depth based on the objective of the study. For instance, in the case of genetic mapping, 0.5–1.0X coverage is sufficient; however, for GWAS, 10–15X coverage can be used. Several NGS platforms can be used for generating WGRS data, such as Illumina Hi-seq (read length of 150–250 bp), PacBio (10–25Kb), and NanoPore (read size of 500 bp to 2.3 Mb). Large LD blocks (several hundred kilo–base pairs) in plants, specially self-pollinating. Large LD blocks include several CGs. Therefore, with dense genotyping, we can have SNP variants in each of the CGs in the block and individual CGs can be identified using GWAS carried out on WGRS genotyping data. A gene for salinity tolerance *Glyma03g32900*, using sequencing data on 106 soybean diversity panels and the SNP-based KASP markers, was developed to improve salinity tolerance in soybean (Patil et al., 2016). Recently, 2,980 chickpea accessions are sequenced to discover 3.94 million SNPs, phenotyping data on 16 traits was used for GWAS analysis and identified 205 SNPs associated with 11 traits, and the associated SNPs were in the genomic regions of 79 CGs playing a role in controlling key traits like seed weight (Varshney et al., 2021).

## Alleviating the phenotyping bottleneck

In the era of different omics like genomics, transcriptomics, and proteomics with the help of NGS technologies, genotyping of large germplasm at multiple locations has become feasible for plant scientists. Thus, phenotyping these large germplasms/populations with higher accuracy have become difficult. Thus, high-throughput genotyping technologies have shifted the bottleneck of plant science from genotyping to phenotyping (Mir et al., 2019). Thus, it has become the need for time to develop high-throughput phenotyping (HTP) approaches (Mir et al., 2019). Several advanced artificial intelligence–based HTP platforms have been developed for crops like rice, maize, and Arabidopsis (Yang et al., 2020). Still, a lot of improvement is required in HTP, which can record multiple phenotypic traits in less time and manpower, which can be associated with large genotypic data of large populations (Mir et al., 2019). The major limitation in phenotyping is recording the multiple traits (agronomic traits, physiological traits, and stress-related scoring) data of large populations at multiple locations in several replications (Furbank and Tester, 2011). There are a lot of chances for error in phenotypic data when recorded manually, and less accuracy leads to false significant associations with molecular markers and wrong interpretation of alleles and genes. HTP is a non-destructive data recording method that allows the plant scientist to increase the size of the experiment by the number of genotypes or replication, or locations (Awlia et al., 2016). PHENOPSIS was one of the first automated imaging and weighing systems developed in Arabidopsis to estimate its response to water deficiency (Granier et al., 2006). However, it has its limitations. HTP platforms are of two types, i.e., HTP platforms for greenhouse or laboratory experiments and open field experiments (Shafiekhani et al., 2017). Although, HTP technologies have been used successfully for genetic dissection of agronomic traits in major field crops like rice, maize, wheat, barley, and brassica (Zhang et al., 2017a; Shi et al., 2013; Yang et al., 2014; Muraya et al., 2017; Topp et al., 2013; Tanabata et al., 2012). The use of these HTP platforms in legume crops is yet to be evaluated at the large fields, population, and multiple location levels (Zhang et al., 2021). A handful of studies has been conducted on legumes such as pea, soybean, and chickpea using a HTP approach for biotic and abiotic stress (Zhang et al., 2012; Friedli et al., 2016; Humplík et al., 2015). Zhang et al. (2021) used the quadcopter unmanned aircraft vehicle multispectral imaging data to predict the yield of chickpea and dry pea with a multivariate regression model. Humplík et al. (2015) used the automatic red blue green image analyzing software in pea to estimate the shoot biomass and photosynthetic activity for cold tolerance. Friedli et al. (2016) used the yerrestrial 3D laser scanning system in soybean for canopy-related traits.

## Advanced methods and tools for GWAS

GWAS has continuously expanded in the last few decades due to advancements in sequencing technologies and the collective effort of the research community. In addition, HTP technologies have allowed us to measure many plant traits that are now frequently analyzed through GWAS tools. Recent years have seen GWAS methods solving issues of computation complexity or enhancing statistical power. It is utilized to detect new associations with traits of interest and to replicate loci detected by other different approaches. A diverse set of researchers is involved in rare-variant detection, statistical model optimization, synthetic associations, and using GWAS findings to better our knowledge of disease etiology. These methods can detect genetic variants associated with biochemical or agronomic and molecular phenotypes. In the future, this will enhance the utility of GWAS methods and their implications for plant science.

### Naïve methods

In the GWAS, linear or logistic regression models are used to test for associations. The linear model is used for continuous traits such as plant height, whereas logistic regression models are used for binary traits indicating that the disease is present or absent. In addition, some covariates are included to account for confounding effects from demographic factors. However, naïve approaches often suffer from inflated false-positive rates that might be induced due to genetic relatedness among study participants (Oetjens et al., 2016). In GWAS, usually, diverse populations are selected, which often have related individuals, making subpopulations within the population. This might lead to spurious associations between SNPs that are more common in each subpopulation and phenotypes of interest if the phenotype has a higher prevalence in that subpopulation.

### Mixed linear model methods

The MLM frameworks used in GWAS have drastically decreased the false-positive rates in comparison with conventional naïve approaches. Among these, the fast GWA tool is an ultra-efficient tool for MLM-based GWAS analysis of biobank-scale data (Jiang et al., 2019). MLM approaches resolve the issue of genetic relatedness among individuals following correction at two levels. These refer to population structure and kinship (Yu et al., 2006). At the first level, the population structure is inferred using genotype data with STRUCTURE tool (Pritchard et al., 2000) or through principal component analysis (Price et al., 2006). The kinship matrix is used at the second level to estimate inter-individual relatedness using the genotype data (Yu et al., 2006). In recent years, many methods have been developed to efficiently solve MLM equations. For instance, a recently available method referred to as EMMA (efficient mixed-model association) provided superior computational speed by eliminating the duplicate matrix operations at each iteration while estimating the likelihood function (Kang et al., 2008). MLM-based methods become computationally intensive for large numbers of samples. The FaST-LMM solves this issue but requires that the number of SNPs be less than the number of samples to derive kinship. The SUPER (Settlement of MLM Under Progressively Exclusive Relationship) method has been developed to extract a subset of SNPs and use them in FaST-LMM to increase the statistical power. Moreover, the compress MLM (CMLM) and enriched CMLM (ECMLM) methods are available for kinship optimization. The modified MLM method called multiple-locus linear mixed model (MLMM) incorporates multiple markers simultaneously as covariates in a stepwise MLM to partially remove the confounding between testing markers and kinship. Furthermore, a new method referred to as fixed and random model circulating probability unification (FarmCPU) completely removes the confounding by dividing MLMM into a fixed-effect model and a random-effect model and using them iteratively. The FarmCPU can analyze the dataset with half million individuals and half million markers within 3 days. However, the random-effect model is computationally intensive in FarmCPU. The new method called Bayesian information and linkage disequilibrium iteratively nested keyway (BLINK) replaces the random-effect model with the fixed-effect model by using Bayesian information criteria. This method also replaces the bin method used in FarmCPU with LD information to eliminate the requirement that quantitative trait nucleotides be uniformly distributed throughout the genome. These all methods are summarized in **Table 2**.

**Table 2:** Advanced methods and tools for GWAS.

S.No.	Method	Description	Reference
1.	MLM	At the first level, the population structure is inferred using genotype data with STRUCTURE tool or through principal component analysis. The kinship matrix is used at the second level to estimate inter-individual relatedness using the genotype data.	Jiang et al., 2019
2.	CMLM	Clusters the individuals into groups and fits the genetic values of groups as random effects in the model that improves statistical power compared to regular MLM methods.	Zhang et al., 2010
3.	ECMLM	Calculate kinship using several different algorithms and then choose the best combination b/w kinship algorithms and grouping algorithms.	Li et al., 2014
4.	FaST-LMM	An algorithm for genome-wide association studies (GWAS) that scales linearly with cohort size in both run time and memory use. This method requires that the number of SNPs be less than the number of samples to derive kinship.	Lippert et al., 2011
5.	SUPER	Uses the associated genetic markers referred as pseudo quantitative trait nucleotides instead of all the markers, to derive kinship.	Wang et al., 2014
6.	MLMM	Include multiple markers simultaneously as covariates in a stepwise MLM to partially remove the confounding between testing markers and kinship.	Segura et al., 2012
7.	FarmCPU	Uses a bin method under the assumption that quantitative trait nucleotides are evenly distributed throughout the genome. Completely eliminates the confounding by dividing MLMM into a fixed effect model and a random effect model and using them iteratively.	Liu et al., 2016
8.	BLINK	Replaces the random effect model with the fixed effect model by using Bayesian information criteria. Uses linkage disequilibrium information to eliminate the requirement that quantitative trait nucleotides be uniformly distributed throughout the genome.	Huang et al., 2019

### Machine learning methods

Recent years have seen tremendous growth in the machine learning methods targeted for GWAS. The approaches used by these methods include classification, regression, ensemble-based learning, and neural networks.

## Regression

Logistic regression coupled with the least absolute shrinkage and selection operator (LASSO) regularization approach is a famous method for GWAS. The penalized logistic regression method was used for the classification of patients with Crohn’s disease using genotyping data at the genome-wide level. The LASSO and ridge regression are among the most frequently utilized penalized regression algorithms (Tibshirani et al., 1996; Hoerl et al., 1970). Recently, a faster and more powerful algorithm was developed by binning the closely occurring SNPs based on LD (An et al., 2020). In addition, the SNPs and phenotypes were mapped using LASSO regression in this method. This method was found to provide a reduced type 1 error rate in comparison with regular MLM and LASSO. To discover variations closely associated with the duloxetine response, some researchers used the standard genome-wide logistic regression (Maciukiewicz et al., 2018). In addition, they extracted the top predictors using LASSO regression. In another study, a preconditioned random forest regression was used to predict late genitourinary toxicity after radiotherapy. This preconditioning involved usage of logistic regression for making a continuous surrogate outcome from the original binary outcomes, which were followed by random forest regression where the surrogate outcome is utilized as a target for prediction. In this study, five-fold cross-validation was conducted for testing the model stability against existing baseline models (Lee et al., 2018). The major drawback of regression approaches is the failure to find higher-order non-linear SNP interactions involved in susceptibility to diseases. The process developed by Zhang and coworkers (2012) utilizes prior information from proteomics and biological pathways for SNP groups. To find the top predictive SNP groups, they used linear regression standardized by group sparse constraint. In the end, group LASSO was used for the regularized linear regression (Yuan et al., 2005). Thus, this approach overcomes the limitations of the regular MLM used in GWAS.

## Classification

Support vector machine (SVM)–based classification methods such as COMBI have been developed for unknown phenotype prediction for a given unseen genotype (Mieth et al., 2016). In this approach, the SNPs having larger SVM weight are chosen, and the remaining SNPs are removed. Next, a chi-squared test is performed, and SNPs that exhibit a p-value below the significant criterion are taken into consideration for intensive study. The SVM method separates labeled data points into two groups with a large difference between them. Some authors proposed using SVM for genetic risk prediction (Mittag et al., 2012). This method has been used for genome-wide risk profiling for diseases such as type 1 diabetes and Parkinson’s disease. In this algorithm, model training is performed using SNP data, which is followed by binary classification of the test dataset. Another researcher used the K-nearest neighbor learning algorithm for the classification of individuals into breast cancer positive and negative groups using their SNPs (Hajiloo et al., 2013). They used a leave-one-out cross validation strategy and external holdout methods for evaluating the performance of their classification algorithm.

### Ensemble learning methods

These methods comprise an ensemble of decision trees. For example, random forest is an example of the ensemble learning algorithm. A bootstrapped subsample of the initial training dataset is used to create each decision tree in this instance. Some authors used gradient-boosting and random forest approaches to identify potent SNPs (Dorani et al., 2018). Nguyen et al. (2015) used a random forest method for selecting informative SNPs. They used a two-stage quality-based approach in model learning for the selection of informative SNPs. This method seems quite useful for the high-dimensional GWAS data. They also used five-fold cross-validation for assessing the potential of the model on different GWAS datasets. In addition, gradient boosting of decision trees was used for GWAS datasets. Others proposed using the XGBoost model for SNP selection (Behravan et al., 2018). This model could be used as an alternative to polygenic risk scoring. In addition, SVM classifier is used at the backend for SNP classification. Using principal component analysis and logistic regression, Oh et al. (2017) suggested a preconditioned random forest regression that converts a binary variable into a continuous variable. Later, Lee and a group of researchers (2020) used this preconditioned model for predicting the risk of breast cancer.

### Neural network-based methods

Liu et al. (2019) developed a convoluted neural network (CNN) model for phenotype prediction using the SNP dataset. Moreover, they applied a saliency map for the first time to choose significant SNPs from training model. They also compared them with statistical methods such as best linear unbiased prediction and Bayesian ridge regression (BRR). In this study, association analysis was performed for quantitative traits of soybean and SNP datasets. Some authors found that increasing the hidden neuron’s number does not affect the performance of the classification model for the case-control settings (Romagnoni et al., 2019). In a different study, authors compared the deep mixed model constituted of CNN and long and short-term memory with standard univariate testing and MLM (Wang et al., 2019).

### Transcriptome-wide association study methods

Transcriptome-wide association study (TWAS) methods perform association analysis for gene expression variations and quantitative traits. TWAS is an approach based on genes with the ability to expand GWAS for a better understanding of functional relationships in complex traits. These methods are alternatives to variant-based association methods representing a subgroup of multi-marker association or locus-based methods. The locus-based methods have been so popular due to the larger apprehension and acceptability of the polygenic framework of the complex traits. In principle, locus-based approaches rely on multiple genetic variants to estimate the contribution of a gene or loci. TWAS uses GWAS results and transcriptome-level information to perform association testing at the gene level (Pividori et al., 2020). The ability to separate and assess the analytical procedures in TWAS simultaneously, provides several opportunities for the development of effective statistical models for the study of gene disease connections.

### PheWAS methods

PheWAS methods perform unique associations in addition to utilizing known genotype–phenotype associations acquired through GWAS. These established relationships might serve as “positive controls” for additional high-throughput analysis. PheWAS methods suffer from high false-positive rates due to thousands of genotype–phenotype associations being tested in such studies (Bastarache et al., 2022). In addition, sample sizes usually also vary across studies impacting the statistical power and the replication among studies. PLATO tool is used to identify associations in PheWAS (Hall et al., 2017). DNAnexus is another tool for genomic analysis that was hosted on Amazon Web Services. This provides a distributed cluster of computers on the cloud allowing much lesser computation time for such studies. With the assistance of the DNAnexus app for PLATO, scatter-process-gather can be used on the platform to train regression models concurrently. This scatter-gather approach initiated multiple AWS virtual machines to simultaneously fit the regression models. Deep-PheWAS is another platform for PheWAS that intertwines quantitative phenotypes from primary care data, disease progression, longitudinal trajectories of quantitative measures, and drug response phenotypes with the composite phenotypes generated from clinically curated data (Packer et al., 2023). Moreover, several tools are available on this platform for efficiently analyzing the association with genetic data under different genetic models.

## GWAS-assisted genomic selection

GS has been utilized as a practical genomic approach for upgrading complex traits in various crops (Thudi et al., 2016; Sandhu et al., 2022). In segregating populations, GS allows identifying lines with higher genomic estimated breeding value (GEBV) using genome-wide marker data. A training population (TP) is used to estimate GEBV, which consists of elite breeding lines and is evaluated for multi-seasons and locations for the target phenotype. Then, a candidate population (CP) is developed by selecting parents based on the GEBVs. GS utilizes all the available genome-wide marker data irrespective of any significant effects. The GS prediction accuracies depend on several factors, including the genome size, ploidy level, interactions between gene and QTL, sample number, relatedness, number and distribution of markers, and model (Yadav et al., 2020). Several statistical methods are used for GS, including Ridge regression best linear unbiased prediction (rrBLUP) and genomic best linear unbiased predictor (gBLUP); both hypothesize a normal distribution of the SNP effects, whereas Bayesian methods like BayesA, BayesB, BayesC, and BayesR allow different variance distributions considering marker effect sizes (Heslot et al., 2012; de Los Campos et al., 2013). On the other hand, kernel approaches help predict non-additive models along with complex multi-environment/trait data (Gianola and van Kaam, 2008; Bandeira E Sousa et al., 2017).

Zhang and coworkers (2016) showed a GWAS-assisted GS with 309 soybean lines and 31,405 SNPs for seed weight using the rrBLUP approach. They showed GS prediction accuracies of 0.75–0.87, outperforming marker-assisted selection with prediction accuracies of 0.62–0.75. Ravelombola et al. (2020) performed a GS approach for soybean cyst nematode tolerance with biomass reduction using 234 soybean accessions in the greenhouse. They used five methods to compute GEBVs, including gBLUP (Zhang et al., 2007), random forest (RF) (Ogutu et al., 2011), rrBLUP (Meuwissen et al., 2001), SVMs (Maenhout et al., 2007), and Bayesian LASSO (Legarra et al., 2011). They found that the prediction accuracies were dependent on the model used, the marker set, and the size of TP. However, the accuracy of GS was higher than the SNPs from GWAS for all selection models and TP sizes.

In alfalfa, Li et al. (2015) used clonal ramets from 185 to 190 individuals for GS of biomass yield across three locations and recorded prediction accuracies of 0.43 to 0.66 for each location. Another study used 322 individual genotypes from 75 genetically diverse alfalfa populations. They tested three Bayesian models (BayesA, BayesB, and BayesC) for 25 agronomic traits, including forage quality traits, dry matter, and fall dormancy (Jia et al., 2018). They reported prediction accuracies of 0.0021 to 0.6485 with no significant differences in the three Bayesian models.

In chickpeas, Roorkiwal et al. (2016) used 320 breeding lines and six different models, including rrBLUP, RF, Bayesian LASSO, BayesB, Kinship GAUSS, and Bayes Cπ for four traits, i.e., seed yield, 100 seed weight, days to maturity, and days to 50% flowering. They reported prediction accuracies ranging between 0.138 (seed yield) to 0.192 (100 seed weight).

Li et al. (2018) showed low prediction accuracies using rrBLUP, Bayesian LASSO, and BRR for grain yield/ha, seed number per plant, 100 seed weight, and early vigor score in chickpea, which can be attributed to the small size of TP.

In common bean, cooking time (CKT), seed weight, and water absorption capacity were evaluated using 922 lines consisting of four populations (a Mesoamerican 8-parental MAGIC population, a biparental RIL, an Andean, and a Mesoamerican breeding line (MIP) panel (Diaz et al., 2020). Six models based on additive effects (BRR, BayesA, BayesB, BayesC, Bayesian Lasso, and gBLUP) and a Bayesian reproducing kernel Hilbert spaces regression (RKHS) models based on both additive and non-additive effects were used. They reported prediction accuracies for CKT ranging from MIP (0.22) to MAGIC population (0.55). A recent study showed prediction abilities ranging between 0.6 and 0.8 were shown in common bean for four agronomic traits under several environmental stresses (Keller et al., 2020)

In peanut or groundnut, 281 Kersting’s groundnut lines were used for GWAS-assisted GSs for several traits, including seed traits, 100 seed weight, leaf length, days to 50% flowering, and days to maturity using 493 SNPs and rrBLUP model (Akohoue et al., 2020). They recorded prediction accuracies ranging from 0.42 to 0.79 for 100 seed weight, seed length and width, days to maturity, and days to 50% flowering. A low prediction accuracy of 0.11–0.20 was reported for traits including plant architecture traits such as height and diameter, petiole length, leaf width, number of seeds, grain yield, number of pods per plant, and number of seeds per pod.

Recently, genomic resources have been made available in some minor legume crops (Varshney et al., 2019; Bohra et al., 2020). In peas, the predictive abilities based on Bayesian LASSO model were 0.28, 0.30, 0.64, and 0.65 for lodging susceptibility, yield, seed weight, and onset of flowering, respectively (Annicchiarico et al., 2019).

**Figure 2:**
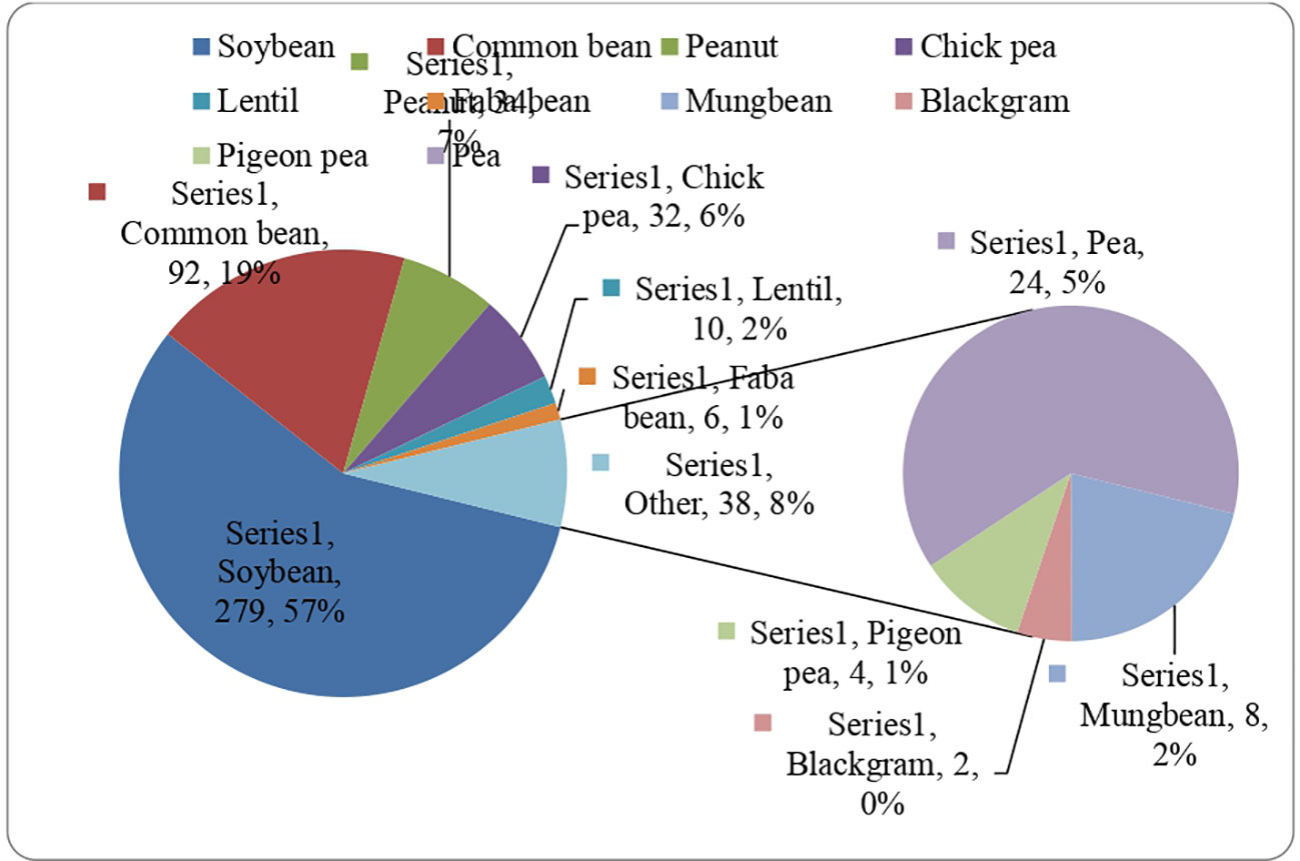
Number of GWAS studies conducted in different leguminous crops from timeline 2012 to 2023.

Several GS methods were used for predicting GEBVs in legume crops (**Figure 2**), but the progress still needs to catch up compared to grain crops, including wheat, rice, and maize. However, the GS approach proved helpful and could be applied in the early stages of legume breeding programs to identify promising progenies and parents based on the predicted breeding values.

## Applications in plant breeding

Since the last decade, GWAS has been successfully used in major legume crops to dissect or identify the genetic bases for various agronomic traits (Bajaj et al., 2015b; Kujur et al., 2015a; Wen et al., 2015; Zhang et al., 2015), quality traits (Hwang et al., 2014; Upadhyaya et al., 2015; Shaibu et al., 2019a), biotic (Butenhoff, 2015; Zhang et al., 2019; Banoo et al., 2020; Zhang et al., 2020; Faridi et al., 2021), and abiotic stress (Thudi et al., 2002; Thudi et al., 2014; Dhanapal et al., 2015; Assefa et al., 2020; Kohli et al., 2020). A plethora of GWASs have been conducted using different types of markers (SSR, SNPs, etc.) in some of the major legumes like soybean (Butenhoff, 2015; Zhang et al., 2015; Zhang et al., 2019; Assefa et al., 2020), chickpea (Kujur et al., 2015b; Upadhyaya et al., 2017; Kohli et al., 2020), groundnut (Zhang et al., 2017c; Shaibu et al., 2019a; Wang et al., 2019), and minor legume crops (Ali et al., 2016; Faridi et al., 2021). Brief details of GWASs conducted in legume crops are given in **Table 1**.

Revolutionization and rapid development in genomic techniques in the recent past have accelerated molecular studies not only in model crops but also in other crops like legumes (Varshney et al., 2005). Sequencing and availability of reference genomes have also made it feasible for the researchers to identify the alleles/QTL association with the desired trait in any germplasm. Although the GWAS approach can be used in any crop with extensive phenotyping and genotyping, it has been used in major legume crops like soybean, chickpea, and groundnut (Mousavi-Derazmahalleh et al., 2019). These major legume crops’ research community has sufficient funding for high-throughput genotyping and phenotyping. As these crops cover a significantly larger area across the globe, significantly diverse and classified germplasms are available for these crops (Mousavi-Derazmahalleh et al., 2019). However, GWAS has its limitations like false-positive association and exclusion of a significant association. All the limitations can be overcome by accurate phenotyping, large enough diverse germplasm, multilocation trials for phenotyping, and accuracy in genotyping. The use of the best suitable model, method, and bioinformatic tools also determines the accuracy of GWAS. The development of model tools for legume crops can trigger the GWAS in major and minor legume crops.

## Conclusion and future perspectives

Legumes are an essential component of human nutrition and play a vital role in sustainable agriculture due to their protein-rich content, soil quality improvement, and reduced environmental impact. With the increasing global population and changing climatic conditions, there is a pressing need to develop high-yielding, disease-resistant legume cultivars that can meet the nutritional needs of the growing population. Improvement in the nutritional and production quality of legume crops with the use of conventional breeding methods is not at the required rate. Whole-genome sequencing is available only for a few major crops. As per the availability, low (RFLP) to high-throughput (SNP) markers have been used in various crops for AM, QTL mapping, or GWAS. NGS has become feasible in the model and even in non-model crops with improved efficiency and affordable sequencing methods. GWAS has been used in major legume crops to identify the genomic region linked with desired characteristics of the plant. It is yet to be exploited in minor legumes with sufficient germplasm/population. The availability of reference genomes and rapid development in genomic techniques has made it feasible for researchers to identify the alleles/QTL association with the desired trait in any germplasm. The use of suitable models, methods, and bioinformatic tools determines the accuracy of GWAS. The development of model tools for legume crops can trigger GWAS in major and minor legume crops. Authentication or precision of identified marker–trait association is required for their utilization in plant breeding programs or MAS/BAC programs. Using NGS and other high-throughput techniques for sequencing will make it possible to develop a genomic-assisted crop improvement program in legumes. Rapid development can be gained concerning agronomic traits, biotic/abiotic stress tolerance, and after-use quality improvement. Legume yield potential is meager compared to other major crops; this yield plateau can be broken in legumes for climate change problems using GWAS with multi-location phenotyping. The integration of these new and improved technologies with traditional breeding methods will help to accelerate the development of new legume cultivars with improved yield and nutritional qualities.

## Author contributions

PS, PK conceptualized the review study and edited the manuscript. PY contributed to advanced methods and tools for GWAS. SS put forth experimental populations for association mapping studies in various crops. GK was associated with GWAS-assisted genomic selection. SSG and MP contributed to new high throughput-genotyping technologies in plants. VS and TT assisted in collecting data for applications of GWAS in plant breeding and drafting the manuscript. All authors contributed to the article and approved the submitted version.
